# Functional Characteristics, Electrophysiological and Antennal Immunolocalization of General Odorant-Binding Protein 2 in Tea Geometrid, *Ectropis* *obliqua*

**DOI:** 10.3390/ijms19030875

**Published:** 2018-03-15

**Authors:** Ya-Li Zhang, Xiao-Bin Fu, Hong-Chun Cui, Lei Zhao, Ji-Zhong Yu, Hong-Liang Li

**Affiliations:** 1 Hangzhou Tea Research Institute, China Coop., Hangzhou 310016, China; zhangyali230@126.com; 2 Zhejiang Provincial Key Laboratory of Biometrology and Inspection & Quarantine, College of Life Sciences, China Jiliang University, Hangzhou 310018, China; eoblfuxiaobin@126.com (X.-B.F.); cjluzl@126.com (L.Z.); 3 Tea Research Institute, Hangzhou Academy of Agricultural Sciences, Hangzhou 310024, China; chc1134@126.com (H.-C.C.); hchyu@126.com (J.-Z.Y.)

**Keywords:** *Ectropis obliqua*, general odorant-binding proteins (GOBPs), ligand-binding assays, electroantennography (EAG), molecular docking, antennal immunolocalization

## Abstract

As one of the main lepidopteran pests in Chinese tea plantations, *Ectropis* *obliqua* Warren (tea geometrids) can severely decrease yields of tea products. The olfactory system of the adult tea geometrid plays a significant role in seeking behaviors, influencing their search for food, mating partners, and even spawning grounds. In this study, a general odorant-binding protein (OBP) gene, *EoblGOBP2*, was identified in the antennae of *E. obliqua* using reverse transcription quantification PCR (RT-qPCR). Results showed that *EoblGOBP2* was more highly expressed in the antennae of males than in females relative to other tissues. The recombinant *Eobl*GOBP2 protein was prepared in *Escherichia coli* and then purified through affinity chromatography. Ligand-binding assays showed that *Eobl*GOBP2 had a strong binding affinity for some carbonyl-containing tea leaf volatiles (e.g., (*E*)-2-hexenal, methyl salicylate, and acetophenone). Electrophysiological tests confirmed that the male moths were more sensitive to these candidate tea plant volatiles than the female moths. Immunolocalization results indicated that *Eobl*GOBP2 was regionally confined to the sensilla trichoid type-II in the male antennae. These results indicate that *Eobl*GOBP2 may be primarily involved in the olfactory activity of male *E. obliqua* moths, influencing their ability to sense tea leaf volatiles. This study provides a new perspective of insect GOBPs and implies that olfactory function can be used to prevent and control the tea geometrid.

## 1. Introduction

Insects sense semiochemicals emanating from host plants through their olfactory systems [1]. This plays an important role in the survival and reproduction of insect species in the natural environment, influencing behaviors such as feeding, courtship, and locating spawning sites [2]. Olfactory systems of insects are mainly composed of soluble binding proteins, olfactory receptors, and odor-degrading enzymes [3]. Significantly, soluble odorant-binding proteins (OBPs) (mainly existing in the antennal sensillar lymph of insects) are involved in odor reception and are thought to carry lipophilic odorants to the olfactory receptor cells through hydrophilic surroundings [4,5].

The first insect OBP was found in *Antheraea polyphemus* in 1981 [6]. Since then, an extremely large number of OBP homologues have been identified from different insect species [7,8]. Insect OBPs are small globulins composed of a single polypeptide chain of about 140 amino acids, characterized by six cysteines that form three pair disulfide bridges at conserved positions [9,10]. According to amino acids’ sequence homologies, distribution patterns, and binding ligand category, insect OBPs are generally divided into three subfamilies, including general OBPs (GOBPs), pheromone-binding proteins (PBPs), and antennal specific proteins (ASPs) [11].

GOBPs are always expressed in the antennae of both sexes, or predominantly found in female antennae. Therefore, they are assumed to bind semiochemicals other than sex pheromones [12]. However, there is significant evidence that GOBPs can bind with pheromone components [13,14,15,16,17]. So, although current evidence indicates that GOBPs exhibit diverse odor sense functions, the actual physiological function of GOBPs in insect species may need further investigation.

The tea geometrid *Ectropis obliqua* (Prout) (Lepidoptera: Geometridae) is one of the most destructive pests of tea plants. Its larvae prefer to feed on tea leaves and severely affect the yield and quality of tea products across agricultural areas in southern and eastern China [18]. Currently, management of *E. obliqua* primarily depends on pesticide application and viral infection introduction [19,20]. However, pesticide residues can endanger human health and pollute non-target environments, and virus infections are slow and limited to larval stages. Therefore, it is imperative to develop new methods and strategies for controlling and managing *E. obliqua*. New strategies that have been proposed involve the use of insect olfactory mechanisms and non-pesticide applications as part of a comprehensive pest management plan [21,22].

To date, only two PBPs have been sequenced in tea geometrids. Both are from the Japanese giant looper moth, *Ascotis selenaria cretacea* [23]. By using transcriptome analysis, some other PBPs have also been recently found in the legs [24], and whole bodies of third-instar *E. obliqua* larvae [25], and in the antennae of *Ectropis grisescens* [26]. However, limited studies have focused on characterizing the olfactory system and functional characteristics of *E. obliqua*. Therefore, we characterized the tissue expression profile of *EoblGOBP2* in *E. obliqua* and the biochemical functions associated with binding of tea plant volatiles (e.g., (*E*)-2-hexenal, methyl salicylate, and acetophenone). Electroantennograms (EAG) were used to test and compare the physiological responses of male and female adult antennae to the candidate plant volatiles. Some of the characteristics of *Eobl*GOBP2 we discovered provide a basis for further understanding the olfactory system function of *E. obliqua* and for the development of non-pesticide control measures.

## 2. Results

### 2.1. Identification and Sequence Analysis of EoblGOBP2

**Figure 1:**
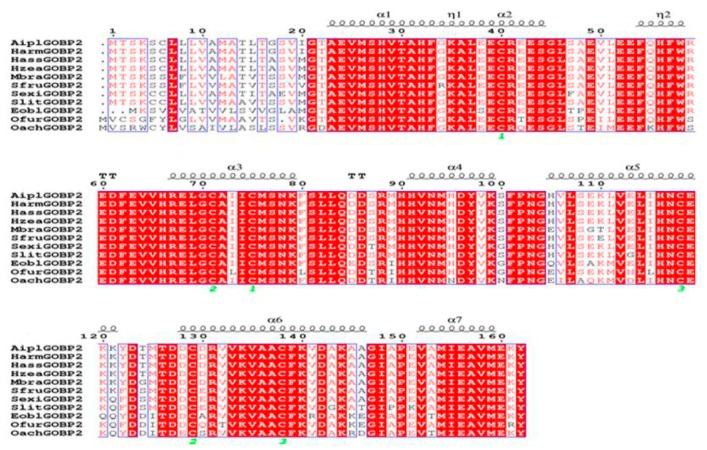
Multiple sequence alignment of 11 GOBP2 homologous proteins sequences. Green numbers represent six conserved cysteines in the GOBP2s. Strictly identical residues are highlighted in white letters with red background. Residues with similar physicochemical properties are shown in red letters. Alignment positions are framed in blue if the corresponding residues are identical or similar. The secondary structure elements for GOBP2s are shown on the top of the sequences; α-helices are displayed as wave line.

**Figure 2:**
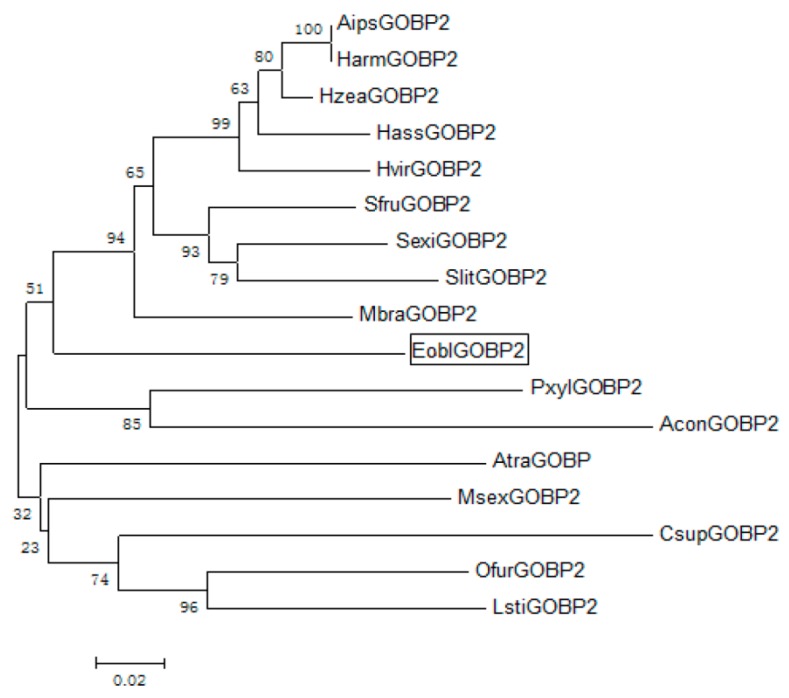
Phylogenetic tree of GOBP2 amino acid sequences in lepidopteran insects. *Eobl*GOBP2 is indicated in box. *Aips*GOBP2 (*Agrotis ipsilon*)/*Harm*GOBP2 (*Helicoverpa armigera*)/*Hzea*GOBP2 (*Helicoverpa zea*)/*Hass*GOBP2 (*Helicoverpa assulta*)/*Hvir*GOBP2 (*Heliothis virescens*)/*Sfru*GOBP2 (*Spodoptera frugiperda*)/*Sexi*GOBP2 (*Spodoptera exigua*)/*Slit*GOBP2 (*Spodoptera litura*)/*Mbra*GOBP2 (*Mamestra brassicae*)/*Pxyl*GOBP2 (*Plutella xylostella*)/*Acon*GOBP2 (*Argyresthia conjugella*)/*Atra*GOBP2 (*Amyelois transitella*)/*Msex*GOBP2 (*Manduca sexta*)/*Csup*GOBP2 (*Chilo suppressalis*)/*Ofur*GOBP2 (*Ostrinia furnacalis*)/*Lsti*GOBP2 (*Loxostege sticticalis*).

The full-length *EoblGOBP2* sequence was amplified from *E. obliqua* cDNA isolated from adult antennae, cloned, and sequenced. The *EoblGOBP2* cDNA sequence contained a 483-bp open reading frame (ORF). The predicted molecular weight of *Eobl*GOBP2 was 18 002.75 D with an isoelectric point of 5.32. Amino acid sequence alignments of *EoblGOBP2* with other GOBP2 homologues in lepidopteran species are shown in Figure 1. Through homologue comparison, we found that *Eobl*GOBP2 had between 61.9 and 98.1% homologies to other GOBP2s. As seen in Figure 1, both *Eobl*GOBP2 and other GOBP2 homologs contain highly divergent signal peptide regions. They also each have six conserved cysteine residues (forming the three disulfide bridges) that followed a common pattern: X_18_-Cys-X_30_-Cys-X_3_-Cys-X_42_-Cys-X_8–10_-Cys-X_8_-Cys-X_24–26_, where X represents any amino acid. This indicates that *Eobl*GOBP2 is a member of the insect GOBP family. A phylogenetic tree, constructed based on the Neighbor-Joining method [27], confirmed the homology between *Eobl*GOBP2 and other insect GOBP2s (Figure 2).

### 2.2. Tissue Expression Profile of EoblGOBP2

**Figure 3:**
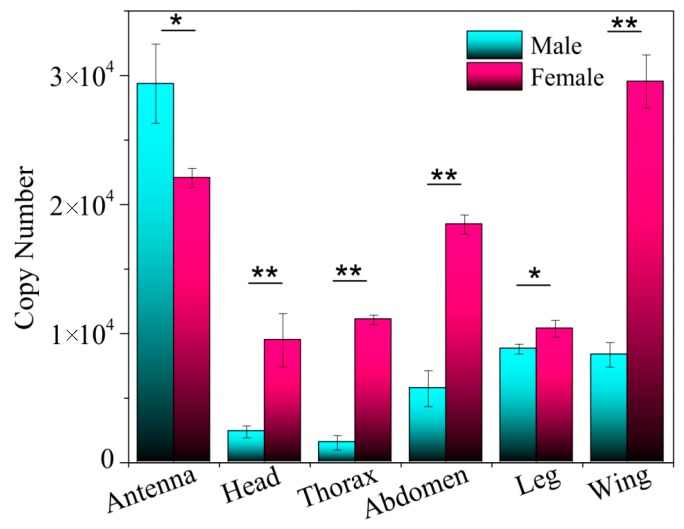
Expression profiles analysis of *EoblGOBP2* in various tissues of male and female *E. obliqua* moths analyzed by the standard curve qPCR method. Significant differences were analyzed by *t*-test (* *p* < 0.05; ** *p* < 0.01)*.*

Based on the absolute quantification method, the expression profile of *EoblGOBP2* in different tissues of both male and female tea geometrids was determined by qPCR. The copy number of *EoblGOBP2* in each tissue was calculated from a standard curve of *EoblGOBP2* plasmid templates. As shown in Figure 3, *EoblGOBP2* was expressed in all tissues and in both sexes. Expression levels were significantly higher in the antennae than in other tissues (excluding the female wings). Notably, *EoblGOBP2* expression was in the male antennae than in the female antennae (*p* < 0.05, *t*-test), but for all other tissues *EoblGOBP2* expression was higher in females than in males (*p* < 0.01, only <0.05 in leg, *t*-test).

### 2.3. Preparation and Purification of Recombinant EoblGOBP2 Protein

**Figure 4:**
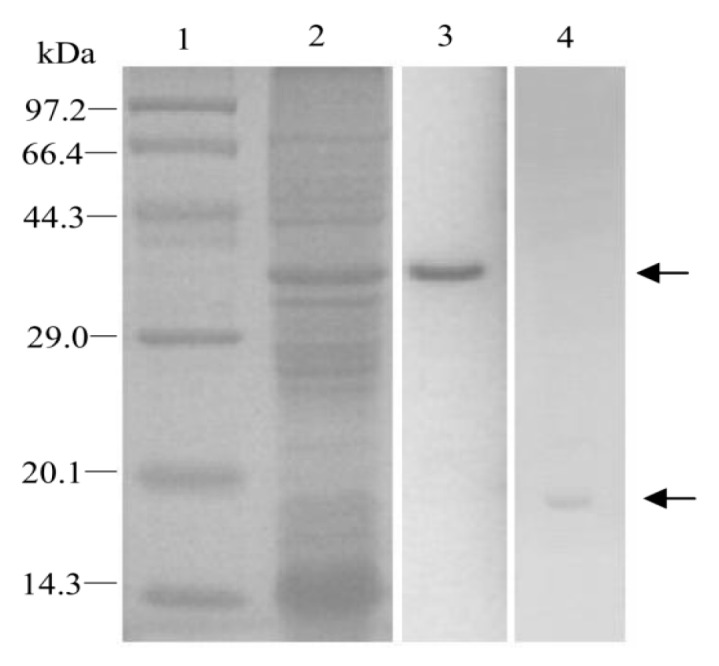
Preparation and purification of *Eobl*GOBP2 protein analyzed by SDS-PAGE. M: protein molecular weight marker. Lane 1: the crude bacterial extracts before induction by IPTG; lane 2: the supernatant of crude bacterial extracts after the induction; lane 3: the purified *Eobl*GOBP2 with His-tag; lane 4: the final purified *Eobl*GOBP2 protein digested by enterokinase.

After *EoblGOBP2* was subcloned into the prokaryotic expression vector pET32a(+), *Eobl*GOBP2 recombinant protein was induced to express in BL21(*DE3*) *E. coli* cells. After bacterial lysis, recombinant protein was isolated from inclusion bodies in the pellet. To separate recombinant proteins, inclusion bodies were redissolved in a high concentration of urea and separated using a Ni^2+^–NTA agarose affinity gel column (Figure 4, Lane 3). The urea concentration was then reduced to allow recombinant proteins to renature. Proteins were subsequently digested by enterokinase and purified again using the Ni^2+^–NTA agarose affinity gel column. The final purified recombinant proteins were subsequently used in ligand-binding assays (Figure 4, Lane 4).

### 2.4. Competitive Fluorescence Ligand-Binding Assay

**Figure 5:**
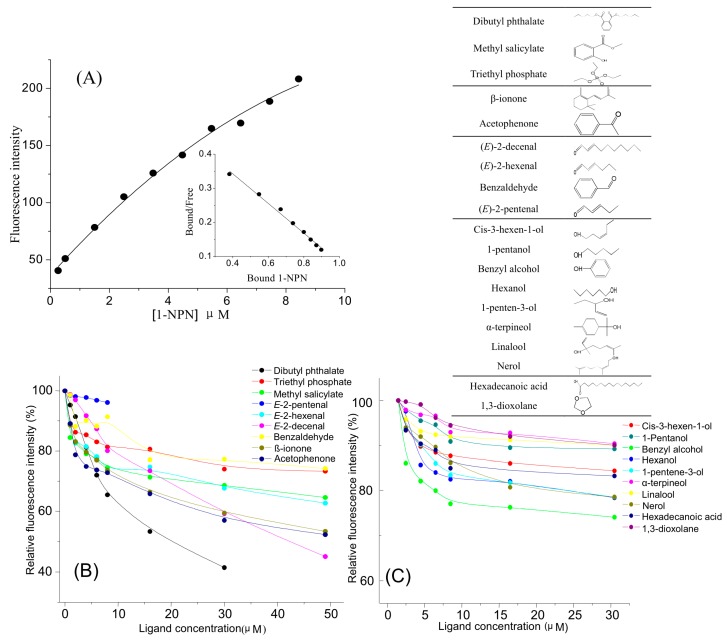
Competitive fluorescence ligand-binding assay of *Eobl*GOBP2 to tea leaf volatiles. (**A**) Binding curve of 1-NPN to *Eobl*GOBP2 and relative Scatchard plot. (**B**,**C**) Competitive binding curves of different tea leaf volatiles to *Eobl*GOBP2. The binding data of all ligands tested were calculated and are listed in Table 1.

For *Eobl*GOBP2 binding assays, 1-NPN was used as a competitive fluorescent reporter. When 1-NPN was added dropwise to the protein solution, the maximum emission peak at 340 nm was shifted to about 420 nm. The dissociation constant of 1-NPN to *Eobl*GOBP2 was approximately 2.31 μM calculated using the Scatchard equation (Figure 5A). Using 1-NPN as a fluorescent probe, the binding affinities of *Eobl*GOBP2 to 19 ligands were measured using competitive binding assays. Competitive binding curves are shown in Figure 5B,C. IC_50_ values and dissociation constants (*K*_D_) were then calculated (Table 1). Results showed that *Eobl*GOBP2 had strong binding affinities to seven carbonyl compounds, including dibutyl phthalate (4.35 μM), methyl salicylate (43.51 μM), β-ionone (12.59 μM), acetophenone (12.97 μM), (*E*)-2-hexenal (32.24 μM), (*E*)-2-decenal (20.72 μM) and benzaldehyde (44.21 μM). However, all of the tested alcohols, one long chain carboxylic acid (hexadecanoic acid), and one heterocyclic compound (3-dioxolane) could not bind with *Eobl*GOBP2.

### 2.5. Molecular Docking and Interaction Analysis

**Figure 6:**
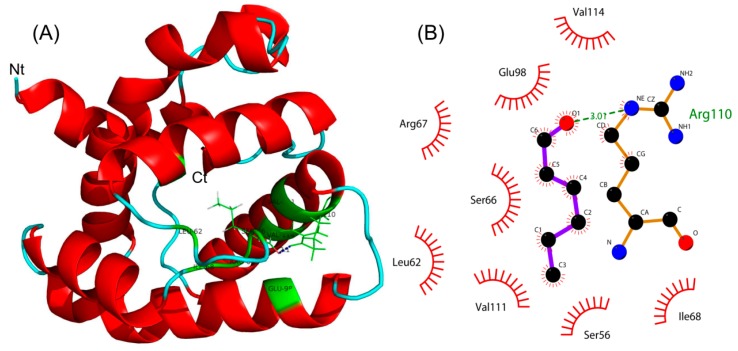
Molecular docking of *Eobl*GOBP2 with (*E*)-2-hexenal. (**A**) 3D structure of *Eobl*GOBP2 with (*E*)-2-hexenal. Red represents α-helix of *Eobl*GOBP2 and green represents the location where *Eobl*GOBP2 interacts with (*E*)-2-hexenal; (**B**) 2D interaction between *Eobl*GOBP2 and (*E*)-2-hexenal. The key amino acid Arg110 of *Eobl*GOBP2 and the hydrogen bond interacting with (*E*)-2-hexenal are shown.

Using the homology modeling from Swiss-Model Workspace [28], the 3D crystal structure of *Eobl*GOBP2 was determined based on the homologous protein template, GOBP2 from *Bombyx mori* (PDB ID: 2wck) [16]. Sequence homology reached 75.89% and global quality estimation score (GMQE) was 0.93. The latter indicates that the constructed *Eobl*GOBP2 model is of high quality (approaching 1.0) and acceptable. The 3D structure of (*E*)-2-hexenal was downloaded from PubChem (CID: 5281168). When the 3D structure of (*E*)-2-hexenal was docked into the predicted optimal binding cavity of *Eobl*GOBP2, the lowest negative energetic value of MolDock was considered to be the optimal binding pose for the *Eobl*GOBP2-(*E*)-2-hexenal complex (Figure 6A). Moreover, (*E*)-2-hexenal was located in one region composed of nine amino acid residues, including four hydrophobic residues (Leu62, Ile68, Val111, and Val114), two polar neutral residues (Ser56 and Ser66), two alkaline residues (Arg67 and Arg110), and one acidic residue (Glu98). We found that a hydrogen bond could be formed between Arg110 (α-nitrogen atom) in the binding cavity and (*E*)-2-hexenal (oxygen atom) (with a total Δ*G* of −9.57 kJ·mol^−1^) (Figure 6A,B). Thus, the observed binding interaction between *Eobl*GOBP2 and (*E*)-2-hexenal could be described and explained by the docking results (e.g., (*E*)-2-hexenal binding was actually promoted by hydrogen bonding and influenced by various kinds of amino acids).

### 2.6. Electroantennograms

**Figure 7:**
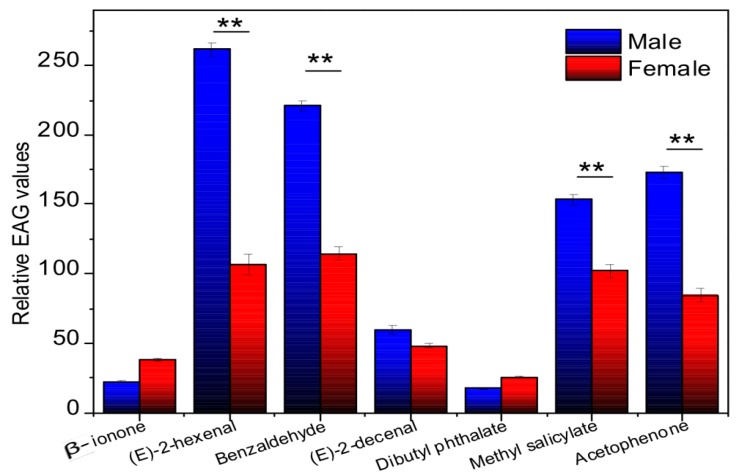
EAG activity of male and female *E. obliqua* antennae to different tea leaf volatiles (10 mg/mL). 1 β-ionone; 2 (*E*)-2-hexenal; 3 Benzaldehyde; 4 (*E*)-2-decenal; 5 Dibutyl phthalate; 6 Methyl salicylate; 7 Acetophenone. ** extremely significant difference (*p* < 0.01, *t*-test).

To characterize the relationship between ligand-binding and *E. obliqua* olfactory activity, seven ligands with strong *Eobl*GOBP2 binding affinities were selected for EAG measurements. EAG values indicated that all candidate volatiles could elicit electrophysiological responses in the antennae of both male and female *E. obliqua*. As can be seen in Figure 7, the response of five compounds, (*E*)-2-hexenal, benzaldehyde, methyl salicylate, acetophenone, and (*E*)-2-decenal, was stronger in male antennae than in female antennae, and this difference was statistically significant (*p*-value < 0.01, *t*-test) for first four compounds. This indicates that *Eobl*GOBP2 may be involved in the antennal odor sensing ability of male *E. obliqua* for some tea plant volatiles. However, this did not extend across all tea plant volatiles as the other two compounds, β-ionone and dibutyl phthalate, only elicited weak responses in male and female antennae.

### 2.7. Immunocytochemical Localization

**Figure 8:**
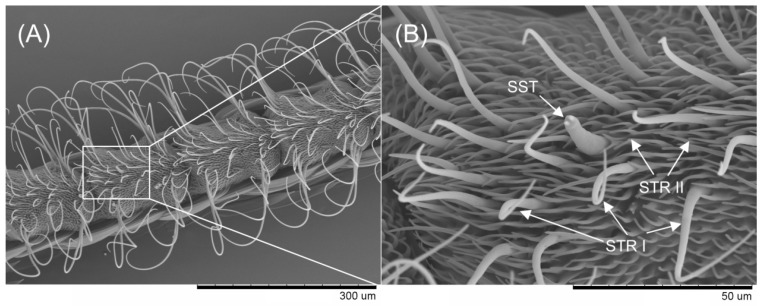
Male *E. obliqua* antennae observed using by SEM. (**A**) Segmental venter of male antennae and (**B**) the significant sensilla were zoomed in and labeled to besensilla trichodea I (STR I), sensilla trichodea II (STR II), and sensilla styloconica (SST), respectively.

To further elucidate the relationship between *Eobl*GOBP2 and olfactory sensing in male moths, we investigated the immunocytochemical subcellular localization of *Eobl*GOBP2 in the male antennal sensilla. From the segmental venter of male antennae observed by SEM, the significant sensilla were mainly found to be sensilla trichodea (including type-I and type-II, STR I and STR II) and sensilla styloconica (SST) (Figure 8). STR I is approximately 150 μm long with a base diameter of about 8 μm. As the most widely distributed sensilla in moth antennae, STR II are short and slender, with a length of about 30 μm and a base diameter of approximately 4 μm. In contrast, SSTs are quite brawny, with lengths of 60 μm and base diameters of about 8 μm.

**Figure 9:**
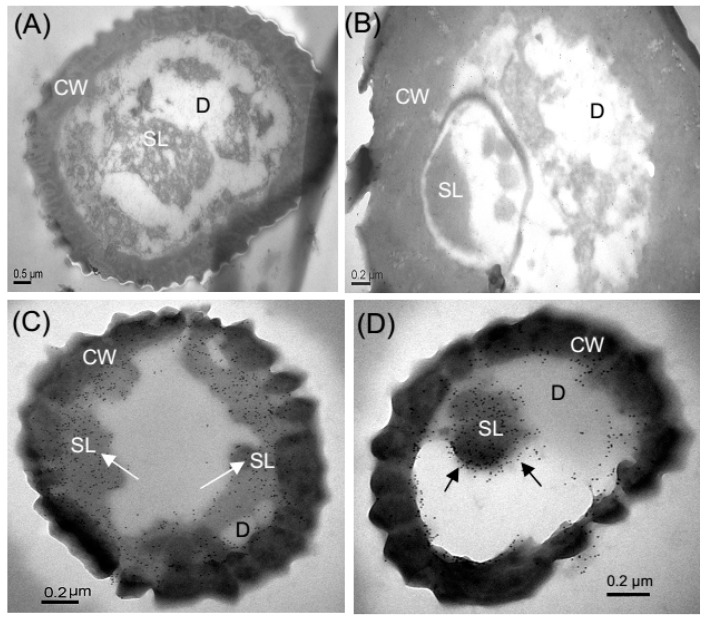
Immunocytochemical localization on antennal sensilla of male moth. *EoblGOBP2* was only found on the sensillar of type s. trichoid II (STR II) (**C**,**D**), rather than s. trichoid I type (STR I) (**A**) and s. styloconicum (SST) (**B**). All kinds of sensilla are confirmed according to the corresponding sensillar diameter observed by SEM in Figure 8. D: dendrite; SL: sensillar lymph; CW: cell wall.

Using the labeled colloidal gold coupled with the corresponding polyclonal antibody, the immunocytochemical localization of *Eobl*GOBP2 was analyzed through the observation of transmission electron microscopy (TEM). As seen in Figure 9, in the antennae of male moths *Eobl*GOBP2 was localized in distinct sections. Indeed, the base diameter, which is about 3 μm, was exactly in the size range of s. trichodea-II STR II (Figure 9C,D), rather than s. trichodea-I (Figure 9A) or s. styloconicum (Figure 9B). The abundance of type AII chemical sensors (e.g., STR II) indicates that *Eobl*GOBP2 is involved in semiochemical recognition processes in male *E. obliqua* moths.

## 3. Discussion

Compared to other moth species, the general odorant-binding protein *Eobl*GOBP2 of the tea geometrid *E. obliqua* was highly structurally similar, with homologies ranging from 61.9 to 98.1% (Figure 2). Phylogenetic analysis indicates that *Eobl*GOBP2 is divided into the same branch as other genes from closely related species including some noctuidae moths (e.g., *Mamestra brassicae*, *Spodoptera litura*, and *Spodoptera exigua*). Moreover, Pyralidae moths (e.g., *L. sticticalis*) and Tortricidae moths (e.g., *Argyresthia conjugella*) were divided into another branch in the phylogenetic tree (Figure 2). This indicates that *EoblGOBP2* is a conservative gene within most of the lepidopteran family.

Expression profile analysis showed that *EoblGOBP2* was highly expressed in antenna of both male and female moths, implying that *EoblGOBP2* may be involved in olfactory-related behavior in adult *E. obliqua.* qPCR results showed that *EoblGOBP2* expression level is higher in male antennae than in female antennae (Figure 3). This is similar to the expression profiles of *LstiGOBP2* of *Loxostege sticticalis* [14], *OachGOBP2* of *Orthaga achatina* [29], and *DtabGOBP1* of *Dendrolimus tabulaeformis* [13]. We postulate that this indicates that male *E. obliqua* are more sensitive to most candidate tea plant volatiles than female moths. EAG assays also support this view (Figure 7). This indicates that *EoblGOBP2* may have higher antennal activities in males compared to females, which may be the result of significant morphological differences between male and female *E. obliqua* antennae [30,31]. However, we found that *EoblGOBP2* was more abundant in other female tissues (e.g., in the wings), which was like *SexiOBP5* in *S. exigua* [32]. This implies that *Eobl*GOBP2 has multiple functions, olfactory and non-olfactory, in the behavior of adult *E. obliqua*.

It has been previously demonstrated using the fluorescence competitive binding assays that GOBP2 homologies can bind to a wind range of tea plant volatiles [13,14,33]. Here, using similar methodologies, *Eobl*GOBP2 was found to bind seven of 19 representative tea plant volatiles [34] with quite high affinities. The seven volatiles can be divided into aldehydes (e.g., (*E*)-2-hexenal, (*E*)-2-decenal and benzaldehyde), ketones (e.g., β-ionone and acetophenone), and esters (e.g., dibutyl phthalate and methyl salicylate), respectively (Figure 5). This is similar to what has been seen with other OBPs. For example, GOBP2 in *Manduca sexta* was shown to competitively bind to “green” and floral odors [35]. Additionally, *Csup*GOBP2 in *C. suppressalis* [33] and three OBP genes in *Drosophila* exceeded the statistical permutation threshold for association with responsiveness to benzaldehyde [36]. Dibutyl phthalate was proven to be one of the best ligands for LUSH of *Drosophila melanogaster* OBP [37]. For β-ionone and (*E*)-2-hexenal, both *Lsti*GOBP2 of *L. sticticalis* [14] and *Alin*OBP10 in *Adelphocoris lineolatus* [38] showed strong binding affinities. Interestingly, none of the candidate alcohols could bind well to *Eobl*GOBP2 (Table 1), although some of them (e.g., benzyl alcohol) could be used as attractants for male *E. obliqua* moths [22]. This means that *Eobl*GOBP2 may contain some distinct binding sites that bind to different kinds of chemical compounds, and that other OBPs present in male *E. obliqua* moths may bind to alcohols in tea leaf volatiles.

As previously mentioned, for most of the five ligands the EAG values of male antennae were higher than in females (Figure 7). This is consistent with what was seen with the sex-specific antennal *EoblGOBP2* expression levels (Figure 3). It has been previously suggested that *Eobl*GOBP2 exhibits more antennal activity in male moths than female moths, which is in accordance with what has been reported in previous *E. oblique* EAG studies [39]. However, EAG only represents the overall activity of all sensilla on the antenna [40]. It could be that other unknown odorant-related proteins are involved in the olfactory recognition of *E. obliqua* except *Eobl*GOBP2. Furthermore, female moths have higher expressional levels in other tissues (except antenna) than males (Figure 3), suggesting that *Eobl*GOBP2 may have other unknown functions (other than olfaction) in female *E. obliqua* moths. 

Generally, PBPs are expressed in the pheromone-sensitive s. trichodea, and GOBPs are expressed in s. basiconica that are thought to be mainly devoted to sensing plant odors [41,42]. For example, GOBP2 has been reported to be mainly expressed in s. basiconica in the male *H. armigera* [43]. Here, we found that *Eobl*GOBP2 was primarily expressed on the type-II s. trichodea, rather than in type-I s. trichodea and or s. styloconicum (Figure 9). According to the morphological similarities between type-II s. trichodea and s. basiconica in other lepidopteran insects [31,44], we conclude that *Eobl*GOBP2 could also be associated with sensing of general plant odors, as demonstrated by the functional experiments above.

Overall, we have found that *Eobl*GOBP2 may have at least two functions, one related to male olfaction (e.g., sensing tea leaf volatiles with particular chemical structures), and the other of unknown function in females (unrelated to olfaction). This expands our understanding about GOBP function in *E. obliqua*. Furthermore, this may assist in the development of a new pest-prevention strategy, that is, odor mixture traps for male tea geometrid moths. This may also enhance the attraction efficiency of the traditional attractants based on the sex pheromones of female tea geometrid moths.

## 4. Materials and Methods

### 4.1. Insects and Tissue Collection

*E. obliqua* larvae were collected from a tea garden close to Daqinggu, Hangzhou, China. Then they were reared in China Jiliang University under conditions of 26 °C, 60–70% RH, and a 12:12 Light:Dark (L:D) photoperiod until eclosion. The fresh male and female moths were first divided into two parts, one part directly for the electroantennography assay and immunolocalization, the other for dissection into six tissues: antennae, heads (without antennae), thoraxes, abdomens, legs, and wings. All anatomical procedures were performed using a sterile scalpel on an ice platform. The excised tissues were immediately immersed in liquid nitrogen and stored at −80 °C for subsequent extraction of total RNA.

### 4.2. Chemical Ligands

*N*-phenyl-1-naphthylamine (1-NPN) (TCI Co., Ltd., Tokyo, Japan) was used as the competitive fluorescent reporter. Nineteen candidate fresh tea volatiles (J&K Chemical Co., Ltd., Tianjin, China) were selected as test ligands. The purity of all ligands exceeded 95%. They are listed in Table S1.

### 4.3. RNA Extraction and Cloning of EoblGOBP2

According to the kit’s manual, total RNA was extracted from the antennae of female *E. obliqua* using TRIzol Reagent (Takara Bio Inc., Kusatsu, Japan). First-strand cDNA was synthesized by the PrimeScript Reverse Transcriptase System (Takara, Japan). Full-length ORF primers of *EoblGOBP2* were designed according to the nucleotide sequence of *EoblGOBP2* (GenBank accession number: ACN29681) using Primer Premier 5.0 software (PREMIER Biosoft International, Palo Alto, CA, USA). The sense primer is: 5′-GTGAATTCATGAAGTCTGTTCTGGTGGCGACGGT-3′ (The *Ec*oR I restriction site is underlined), and antisense primer is: 5′-GGCAAGCTTGCTAATA CTTCTCCATGACAGCTTC-3′ (The *Hin*d III restriction site is underlined). The amplification of *EoblGOBP2* cDNA fragment was performed in a S1000™ Thermal Cycler (Bio-Rad, Hercules, CA, USA) with the following PCR procedure: 95 °C for 4 min; 35 cycles at 94 °C for 30 s, 47 °C for 30 s, and 72 °C for 40 s; final extension at 72 °C for 10 min.

### 4.4. Expression Profiles of EoblGOBP2 in Various Tissues

Using Primer Premier 5.0 software (PREMIER Biosoft International), the primers of qRT-PCR were designed as 5′-CAGAGGCTCGTGAGGATG-3′ (sense primer) and 5′-CCTGACCATTAGGGAAGC-3′ (antisense primer), and synthesized by Corporation Sangon Bietech (Shanghai, China). In qRT-PCR experiments, the absolute quantification strategy based on a standard curve was used to measure the expression level of *EoblGOBP2* in various tissues of *E. obliqua*. The constructed pMD-18T/*EoblGOBP2* plasmid was used as the standard template.

The copy number was calculated based on the formula: Copy number = (Concentration × 6.02 × 10^23^)/(Relative molecular mass ×10^9^). The plasmid was extracted at a concentration of 52 ng/μL. Based on the concentration, the copy number of the standard plasmid was calculated as 7.458 ×10^9^ copies. A 1000-fold diluted plasmid was used as a stock solution for 10-fold serial dilutions. All diluted plasmids were used as a reference template for the acquisition of a standard curve for qRT-PCR.

qRT-PCR was performed in Bio-Rad iQ5 (Bio-Rad, USA). The 20 μL reaction consisted of 10 μL of 2 ×SYBR Premix EX Taq, 0.4 μL each primer (10 μM), 2 μL cDNA sample (total 12 tissues), 6.8 μL ddH_2_O. The PCR program was at 95 °C for 30 s, 40 cycles of 95 °C for 10 s, and 59 °C for 30 s. Three experimental replicates of each sample were performed.

### 4.5. Expression and Purification of Recombinant EoblGOBP2 Protein

The full-length *EoblGOBP2* cDNA was cloned into the bacterial expression vector pET-32a(+). The enzymatic digestion sites are designed as *Eco*R I and *Hin*d III. The recombinant vector pET-32/*EoblGOBP2* was transformed into competent BL21(*DE3*) *E. coli* cells. *E. coli* cells were cultured until the OD_600_ value reached 0.6, while β-d-1-thiogalactopyranoside (IPTG) was added into the LB medium at a final concentration of 1 mM. The bacterial cultures were shaken continuously at 200 rpm at 30 °C for 5 h to induce recombinant *Eobl*GOBP2 protein. After the cultured cells were sonicated, the recombinant protein was detected as an inclusion body by SDS-PAGE in the pellet. Purification was performed using a Ni^2+^-NTA agarose gel column (GE Healthcare, Pittsburgh, PA, USA). The *EoblGOBP2* protein was eluted with increasing concentrations of imidazole (10, 20, 50, 100, 200, 300, and 400 mM) in PBS buffer (pH 7.4). The *EoblGOBP2* protein was refolded with gradient-descending urea dialysis following the method in [45], then digested with enterokinase and subjected to a second purification using the Ni^2+^-NTA agarose affinity gel column. 

### 4.6. Competitive Ligand-Binding Assay

Ligand-binding assays were performed on a RF-5031pc Fluorescence Spectrophotometer (Shimadzu, Tokyo, Japan). The excitation and emission slits were set to 10 nm. Fluorescence spectra were recorded from 300 to 550 nm with an excitation wavelength of 282 nm. The recombinant *Eobl*GOBP2 protein solutions (1.5 μM), served in 1 cm light path quartz cuvette, were titrated with 1 mM 1-NPN solution in methanol. The fluorescence intensity at the maximum emission wavelength of about 328 nm was linearized using the Scatchard equation [46], then the dissociation constants for 1-NPN (*K*_1-NPN_) was calculated. 

Nineteen fresh tea leaf volatiles were used as test ligands. For the binding affinities of the ligands to *Eobl*GOBP2, a mixture of 1-NPN and *Eobl*GOBP2 protein was titrated with 1 μM ligands dissolved in methanol. The IC_50_ values were obtained by data linearization, and the dissociation constants (*K*_D_) were calculated using the following equation: *K*_D_ = [IC_50_]/(1 + [1-NPN]/*K*_1-NPN_) [33,47], where [1-NPN] is the free concentration of 1-NPN, and *K*_1-NPN_ is the dissociation constant of the protein/1-NPN complex.

### 4.7. Molecular Docking

The predicted the 3D crystal structure of *Eobl*GOBP2 from the Swiss-Model Workspace based on the 3D crystal template in the Protein Data Bank (PDB) [28]. The three-dimensional (3D) conformer structure of candidate tea volatile was downloaded from the chemical compound database of PubChem [48]. The binding docking analysis of *Eobl*GOBP2 with compound was performed using Molegro Virtual Docker (MVD 4.2 free trial) software [49]. By using the searching algorithm of MolDock Optimizer, the binding pose of the *Eobl*GOBP2-compound complex was predicted based on the energetic evaluation of the MolDock Score. The optimized 2D and 3D binding poses were then displayed using Ligplot+ [50] and Pymol software [51], respectively.

### 4.8. Electroantennograms (EAGs)

The antennal olfactory responses of *E. obliqua* to seven candidate tea volatiles were tested by the EAG technique. Prior to EAG experiments, all test ligands were dissolved into liquid paraffin at a concentration of 0.1 (*v*/*v*). The fresh worker bee antenna were excised from its base, and immediately attached onto the fork electrode holder using non-drying clay (Spectra 360 Electrode Gel, Parker Laboratories Inc., Orange, NJ, USA). When the signal response to fresh air shows a smooth response, test odor compounds can be used to stimulate the antennae. All antennae should be tested in three replicates.

The odor stimulus was performed in the pulse while recording the electroantennogram of antennae. (*E*)-3-hexenol and liquid paraffin were chosen as the positive and negative control CK, respectively. All antennae were stimulated with five samples, and each tested sample was repeated six times. EAG signals were recorded and analyzed with EAG-Pro software (Syntech, Hilversum, NT). The relative values of EAG response were calculated using the following formula: S_r_ = (S_c_ − CK_m_)/(R_m_ − CK_m_)·100% [52]. Where S_r_ is the relative value of the EAG response, S_c_ is the EAG response for a certain odor, CK_m_ is the mean of the EAG responses of liquid paraffin before and after the test odor stimulus, and Rm is the mean of the EAG response of (*Z*)-3-hexenol.

### 4.9. Scanning Electron Microscopy (SEM)

The male and female antennae of *E. obliqua* were quickly excised from adult heads and immersed in a concentration series of ethanol (50%, 70%, 80%, 90%, 95%, and 100%) for 15 min each. After air drying, the samples were mounted on holders and examined using a SEM of TM-1000 (Hitachi Ltd., Tokyo, Japan) after gold coating with a MC1000 sputter coater (Hitachi, Japan).

### 4.10. Immunocytochemical Localization

The induced purified *Eobl*GOBP2 protein was diluted to the concentration required by the immunization procedure and then mixed with complete Freund’s adjuvant by equal volume and injected into New Zealand white rabbits for immunization. The obtained antibody was determined by ELISA and stored at −20 °C. The fresh male antennae of *E. obliqua* moths were chemically fixed in a mixture of formaldehyde (3%), glutaraldehyde (0.1%), and sucrose (4%) in 0.1 M PBS (pH 7.4) at 4°C for 2–3 h, then dehydrated in an ethanol series and polymerized embedded in K4M (Sigma, St. Louis, MO, USA) and irradiated with UV at −20 °C for 72 h. Ultrathin sections (50–100 nm) were cut with a glass knife on an Ultracut E ultramicrotome (Reichert-Jung, Vienna, Austria). For immunocytochemistry, the nickel grids adhering to ultrathin sections were floated on droplets of the following solutions on parafilm with sequential steps: ddH_2_O, BT (10 mM PBS containing 1% bovine serum albumin and 0.05% Tween20), primary antiserum diluted with BT (dilution 1:100) (unimmunized rabbit serum parallelly used as blank control), 10 mM PBS, secondary antibody that goat anti-rabbit IgG coupled to 10-nm colloidal gold (AuroProbe EM, GAR G10, Amersham Biosciences, Piscataway, NJ, USA) diluted with BT (dilution 1:100), twice on 10 mM PBS, three to five times on ddH_2_O. After being stained by 3% uranyl acetate for 5 min, the results of immunolocalization of *Eobl*GOBP2 were observed and photographed using the transmission electron microscope (TEM) H-7650 (Hitachi, Japan).

## Figures and Tables

**Table 1:** Dissociation constants (μM) of *Eobl*GOBP2 and tea volatiles with the fluorescent probe 1-NPN.

Chemical Category	Tea Volatiles	[IC_50_] (μM)	*K*_D_ (μM) Dissociation Constant	Percentage in the Total Tea Leaves Volatiles (%)
Esters	Dibutyl phthalate	19.42	4.35	0.08
	Methyl salicylate	194.05	43.51	2.37
	Triethyl phosphate	―	―	0.53
Ketones	β-ionone	56.13	12.59	0.11
	Acetophenone	57.86	12.97	0.31
Aldehydes	(*E*)-2-decenal	92.41	20.72	0.23
	(*E*)-2-hexenal	143.08	32.24	0.46
	Benzaldehyde	197.20	44.21	0.13
	(*E*)-2-pentenal	―	―	0
Alcohols	(*Z*)-3-hexenol	―	―	0
	*n*-pentanol	―	―	0.45
	Benzyl alcohol	―	―	0
	*n*-hexanol	―	―	0.06
	1-penten-3-ol	―	―	0.09
	α-terpineol	―	―	0.31
	Linalool	―	―	0
	Nerol	―	―	0.200
Others	Hexadecanoic acid	―	―	0.251
	1,3-dioxolane	―	―	0.435

Solution of protein was at 1.5 μM with and the added concentration of 1-NPN was in line with the dissociation constants of *Eobl*GOBP2/1-NPN complex calculated. Dissociation constants (*K*_D_) were calculated from the values of ligands halving the 1-NPN fluorescence (IC_50_), as described in the methods. Dissociation constants of ligands whose IC_50_ exceeded 50 mM are represented as ‘‘―’’.

## Supplementary Materials

The following are available online at http://www.mdpi.com/1422-0067/19/3/875/s1.
