# Supplementary material for: Functional Characteristics, Electrophysiological and Antennal Immunolocalization of General Odorant-Binding Protein 2 in Tea Geometrid, Ectropis obliqua
Source: Int J Mol Sci. 2018 Mar 15;19(3):875. doi: 10.3390/ijms19030875 (PMC5877736; doi:10.3390/ijms19030875)
Supplement: Supplementary file 1 [file ijms-19-00875-s001.pdf]

Table S1. Chemical ligands

Name	Purity (%)	Name	Purity (%)
Cis-3-hexen-1-ol	＞98	Dibutyl phthalate	＞99
1-pentanol	＞99.5	Triethyl phosphate	＞99
Benzyl alcohol	＞99.5	Methyl salicylate	＞98
Hexanol	＞98	(*E*)-2-pentenal	＞95
1-pentene-3-ol	＞98	(*E*)-2-hexenal	＞95
α- terpineol	＞97	(*E*)-2-decenal	＞95
Linalool	＞98	Benzaldehyde	＞98
Nerol	＞98	β-ionone	＞96
Hexadecanoic acid	＞97	Acetophenone	＞98.5
1,3-dioxolane	＞99	N-phenyl-1-naphthylamine	＞98
